# ‘Why should I start medication when I am not feeling sick?’ A qualitative study of refusal, initiation, completion and discontinuation of tuberculosis preventive treatment among people living with HIV in sub-Saharan Africa

**DOI:** 10.1136/bmjph-2025-004163

**Published:** 2026-07-14

**Authors:** Lindiwe Tsope, Lucy Chimoyi, Audrey Kennar, Israel Rabothata, Solomon Sisay, Kenny Sithole, Nicole Kawaza, Tsitsi Apollo, Chlorata Gwanzura, Solomon Dressie, Ahmed Bedru, Christiaan Mulder, Violet Chihota, Christopher J Hoffmann, Jill Owczarzak

**Affiliations:** 1 Implementation Research Division, Aurum Institute, Johannesburg, South Africa; 2 Johns Hopkins University, Baltimore, Maryland, USA; 3 KNCV Tuberculosis Foundation, Addis Ababa, Ethiopia; 4 Clinton Health Access Initiative, Harare, Zimbabwe; 5 National TB and Leprosy Program, Zimbabwe Ministry of Health and Child Care, Harare, Harare Province, Zimbabwe; 6 National TB and Leprosy Program, Addis Ababa City Administration Health Bureau, Addis Ababa, Ethiopia; 7 Department of TB Elimination and Health System Innovations, KNCV Tuberculosis Foundation, The Hague, Netherlands; 8 Global Science, Aurum Institute, Johannesburg, South Africa; 9 School of Public Health, University of Witwatersrand, Johannesburg, Gauteng, South Africa; 10 Department of Medicine, Vanderbilt University School of Medicine, Nashville, Tennessee, USA; 11 Department of Medicine, Johns Hopkins University, Baltimore, Massachusetts, USA

**Keywords:** HIV, Public Health, Qualitative Research, Preventive Medicine

## Abstract

**Introduction:**

Tuberculosis (TB) preventive treatment (TPT) is effective in preventing TB among people living with HIV (PLHIV). Patient experiences and preferences contribute to optimising the delivery cascade for TPT among PLHIV. We describe patient-level reasons for initiation, completion, refusal and discontinuation of 3HP (high-dose rifapentine (RPT) 900 mg plus isoniazid (INH) 900 mg weekly for 3 months) and INH in Ethiopia, South Africa and Zimbabwe.

**Methods:**

We conducted a total of 141 45–60 min in-depth interviews (IDIs) with adult PLHIV in Ethiopia (n=45), South Africa (n=45) Zimbabwe (n=51) between March and June 2022. Participants were attending routine HIV care in nine healthcare facilities and interviewed using a study-designed interview guide. The guide explored three categories: TB and TPT knowledge, reasons for TPT initiation and experiences of taking TPT and antiretroviral therapy (ART). Recordings were transcribed verbatim, translated and thematically analysed on MAXQDA using an abductive coding scheme incorporating the information-motivation-behavioural (IMB) skills model to understand reasons behind TPT uptake patterns.

**Results:**

Of the 141 participants, 28 were still on TPT, 66 completed, 22 discontinued and 25 refused to initiate TPT. The majority were female (88%, 124/141), with an average of 36 years. IMB constructs were reflected in participants’ TPT uptake patterns, with illustrative quotes highlighting key patterns across the dataset. In relation to information, participants demonstrated knowledge of TPT and its benefits; however, there was confusion about the role of TPT in the absence of TB disease. Prior TB experiences, social support and engagements with healthcare workers were motivations for uptake. The main behaviours found included adoption of adherence strategies and use of medication reminders. Side effects, fears of pill burden and contraindications had negative outcomes for TPT uptake and completion.

**Conclusions:**

The findings underscore the need to enhance the understanding of TPT through targeted interventions at clinic and community levels. Strengthening these efforts may improve TPT uptake and completion rates.

**WHAT IS ALREADY KNOWN ON THIS TOPIC**

**WHAT THIS STUDY ADDS**

- This study provides qualitative insights into the motivations, experiences and challenges of PLHIV regarding TPT uptake, completion, discontinuation and refusal.
- It highlights the influence of pill burden, social support, health system factors and participant perceptions including prior TB experiences on adherence.

**HOW THIS STUDY MIGHT AFFECT RESEARCH, PRACTICE, OR POLICY**

- Findings underscore the need for patient-centred interventions, including clear education on TPT, interactive counselling, peer support and culturally sensitive approaches that integrate community and faith-based engagement.
- Addressing facility-level barriers and misinformation can improve TPT uptake and completion, informing implementation strategies in similar settings.

## Introduction

 Tuberculosis (TB) remains one of the leading causes of death among people living with HIV (PLHIV). In 2023, an estimated 671 000 PLHIV acquired TB, with the highest burden among countries in the WHO’s African Region including Ethiopia, South Africa and Zimbabwe.^1^ The WHO recognises preventing TB infection and stopping progression from infection to disease as critical to reducing TB incidence, hence the recommendation of TB preventive treatment (TPT) for all PLHIV without TB disease.^1 2^

The traditional TPT regimen of daily isoniazid for 6–12 months, reffered to as isoniazid preventive therapy (IPT), has not achieved desired levels of use.^2 3^ Between 2018 and 2022, only 52% (15.5 million) of the targeted 30 million people were provided with TPT (primarily isoniazid). The WHO reported that only 20% of those eligible for TPT completed the entire cascade of care.^4^ Complex social, economic and structural factors shape TPT uptake and completion, including challenges with stock availability, low acceptance by healthcare workers due to concerns regarding toxicity and generating isoniazid resistance, and completion due to long duration of treatment.^5^,^7^ Existing research has largely outlined the barriers and facilitators to the traditional TPT uptake and completion.^8^,^11^ Several individual-level facilitators have been identified, including fears of contracting TB, awareness of TB risk and transmission, willingness to initiate TPT, trust in healthcare workers, ease of regimen and perceived benefits of direct observation therapy or self-administered therapy.^9 11 12^ Some of the key patient-level barriers to uptake include socioeconomic status, insufficient patient education on TPT, pill burden, drug-drug interaction and fear of side effects.^8^,^11^

An alternative to 6–12 months of isoniazid is high-dose rifapentine (RPT) 900 mg plus isoniazid (INH) 900 mg weekly for 3 months (3HP).^13^ This shorter course regimen has demonstrated equivalent efficacy, better tolerability and higher completion rates than isoniazid monotherapy.^1214^,^16^ However, little is known regarding the personal experiences with 3HP. Using a qualitative research approach, and drawing on experiences of individuals, this study sought to generate an in-depth understanding of patient-level reasons for initiation, completion, refusal to initiate and discontinuation of both 3HP and IPT in Ethiopia, South Africa and Zimbabwe.

## Methods

### Study design and setting

This cross-sectional qualitative study was part of a larger prospective study (Opt4TPT) aimed at understanding the TPT care cascade in Ethiopia, South Africa and Zimbabwe. PLHIV were recruited in three primary health facilities in each country: two implementing 3HP and one IPT. In Ethiopia and Zimbabwe, 3HP was prescribed as a fixed dose combination of three pills; in South Africa, patients prescribed 3HP were given 10 pills including pyridoxine.

### Study population and sampling technique

The study population consisted of participants purposively recruited from the main prospective cohort study. Eligible participants were PLHIV aged ≥18 years receiving HIV care at study facilities, either newly initiating or refilling ART. Participants were purposively sampled to ensure representation across four TPT uptake categories at the time of recruitment: (1) completed TPT, (2) currently receiving TPT, (3) discontinued TPT and (4) declined TPT initiation.

Recruitment aimed for up to 15 participants per group per country, with a target of approximately 45 participants per country and 135 participants across the three study countries. Sampling also sought to achieve diversity by sex (1:1 male: female ratio) and age (<35 years vs ≥35 years). Approximately 67 participants were anticipated to be TPT-naïve (refused or not offered TPT), while 68 participants had received TPT (completed or discontinued).

### Patient and public involvement

At the beginning of the study, the investigators introduced the study to relevant stakeholders in the Ministries and Department of Health. During study implementation, the study team frequently met with healthcare workers at facility level and national and regional stakeholders during monitoring visits. We piloted the IDI guides to test and refine the questions and probes with six participants, two in each country. At the end of the study and data analysis, we presented country-specific findings to key stakeholders including Ministries/Department of Health, healthcare workers and policymakers in each of the countries. Patients were not involved in the design, or conduct, or reporting or dissemination plans of our research.

### Conceptual framework and themes explored

We adapted the information-motivation behavioural (IMB) model^17^,^20^ to (1) holistically guide data collection, analysis and interpretation; and (2) to highlight how the IMB constructs influence behaviour, that is, TPT completion, discontinuation and refusal. The IMB model uses three core determinants of initiating and sustaining a behaviour over time^17 19 20^: (1) information, which refers to the facts and knowledge that individuals have about the behaviour, service or treatment; (2) motivation, which refers to the social and personal attitudes, beliefs and motives for engaging in a particular behaviour; and (3) behavioural skills, which refers to the skills and self-efficacy guiding the successful adoption of behavioural patterns. Using the core domains of the IMB model, we were able to explore factors associated with TPT behaviour, that is, TPT initiation and persistence or completion, or TPT non-initiation and discontinuation.

### Data collection

Trained research assistants approached potential participants during scheduled study visits and invited them to participate in the qualitative study. Eligibility lists were generated using clinic records and the parent study database. Written consent was obtained prior to participation, and demographic information was collected.

In-depth interviews (IDIs), lasting between 45 and 60 min, were conducted face-to-face by trained research staff in quiet rooms at the respective facilities. Participants were reimbursed between ~$10 and $25 for their time and travel, in accordance with country-specific guidelines. IDIs were conducted in the participants' preferred languages: Ethiopia (Amharic, English), South Africa (IsiZulu, SeSotho, English) and Zimbabwe (Shona, Ndebele, English). IDIs were audio recorded and transcribed verbatim, and where necessary, interviews were translated into English.

Participants who had completed or discontinued TPT were interviewed as soon as feasible after their treatment status was documented, typically during subsequent clinic or study visits. While efforts were made to minimise delays between treatment outcome and interview, timing varied depending on scheduling and participant availability; the potential for recall bias related to this interval is acknowledged.

All participants were asked the same questions in the same sequence. Coding was conducted after data collection.^21 22^ Data saturation was monitored throughout the data collection process and achieved through regular debriefing discussions with the research assistants to refine probing and assess emerging themes. Thematic saturation was confirmed through iterative meetings with the multidisciplinary team (LT, AK, LC, VC, CJH and JO).^23^

### Data analysis

Project coordinators in each country checked the accuracy of the transcripts against audio recordings. Multiple readings of the transcripts were done by LT, AK and LC to capture the context and manually code and categorise new themes. Transcripts were imported into MAXQDA (a qualitative data management and analysis software program)^24^ to group and organise the key themes across groups.^25^ Transcripts were stored securely on password-protected computers, and audio-recordings were deleted from recorders. Reporting of data adhered to Consolidated criteria for Reporting Qualitative research criteria.^26^

Three members of the study team with PhD and Master’s degree qualifications (LT, KM and AK) used an inductive approach to thematically analyse the data.^27 28^ To develop the initial codebook, at least 10% of the transcripts were coded by the abovementioned three researchers to ensure trustworthiness.^22 27 29^ The initial codebook with emerging themes was discussed with the study investigators (JO and LC). Subsequently, the final codebook was developed, guided by the IMB model and used to code the remaining transcripts.^17^,^20^ We highlight the major themes and subthemes that emerged and use supportive direct quotations from the participants. Themes and subthemes started to converge after 12–15 interviews, and this indicated that saturation had been reached.^23^

### Ethical considerations

We obtained approval from the local district research ethics committees in Ethiopia, South Africa, and Zimbabwe, followed by permission from the participating healthcare facilities. All participants provided written consent for participation, recording of IDIs and use of their direct quotations. We ensured that all participant records and information were anonymised and deidentified before analysis.

## Results

### Participants characteristics

Demographic characteristics of 141 participants (45 each from Ethiopia and South Africa and 51 from Zimbabwe) from 141 IDIs (table 1) conducted between March and June 2022, revealed 28 people still on TPT, 66 completed, 22 discontinued and 25 refused to initiate TPT. Most were female (n=124; 88%), average of 36 years, on ART for more than 5 years (n=81; 57%), and were on IPT (n=60; 43%). Although, age data and TPT regimen data for two participants who had initiated TPT was missing.

**Table 1:** Demographic characteristics of PLHIV participating in IDIs by TPT uptake patterns

Parameter	Completed TPT n (%)	Still on TPT n (%)	Discontinued TPT n (%)	Refused TPT n (%)	Total
Total participants	66 (46.8)	28 (19.9)	22 (15.6)	25 (17.7)	141 (100.00)
Gender					
Male	11	3	1	2	17
Female	55	25	21	23	124
Age (years)*					
18–25	14	0	3	1	18
26–35	14	14	2	6	36
36–45	14	8	11	11	44
45+	17	6	9	9	41
Years on ART					
≤1 year	6	4	2	1	13
>1–5 years	22	9	8	8	47
>5 years	38	15	11	17	81
TPT regimen†					
3HP	33	11	10	–	54
6 hours/12 hours	31	17	12	–	60
Country					
Ethiopia	18	7	8	12	45
South Africa	15	12	8	10	45
Zimbabwe	31	9	5	6	51

* Age data were missing for two participants.

† TPT regimen was applicable only to participants who initiated TPT (completed TPT, still on TPT or discontinued TPT; n=116). Participants who refused TPT did not initiate treatment and therefore had no assigned TPT regimen. Regimen data were missing for two participants who initiated TPT*.*

ART, Antiretroviral therapy; 3HP, high-dose rifapentine 900 mg plus isoniazid 900 mg weekly for 3 months; IDIs, in-depth interviews; PLHIV, people living with HIV; TPT, tuberculosis preventive treatment.

Across the three countries (n=141), 38% of participants completed 3HP and 42% completed IPT, with similar completion rates observed in Ethiopia and Zimbabwe and slightly lower 3HP completion in South Africa. The 3HP regimen differed by country, with South Africa using a higher pill burden (10 pills including pyridoxine) compared with Ethiopia and Zimbabwe, where a 3-pill fixed-dose combination was used (table 2).

**Table:** 2 TPT regimens and completion by country

Country	Total participants (n=141)	3HP n (%)	IPT n (%)	Country-specific 3HP dose regimen
South Africa	45	38	40	10 pills (includes pyridoxine)
Ethiopia	45	31	42	3 pills (fixed-dose combination)
Zimbabwe	51	45	45	3 pills (fixed-dose combination)

3HP, high-dose rifapentine 900 mg plus isoniazid 900 mg weekly for 3 months; IPT, Isonaizid Preventive Therapy; TPT, tuberculosis preventive treatment.

### Theme 1: information

#### Knowledge of TPT and understanding of TPT benefits

All groups of participants demonstrated a certain level of knowledge regarding TPT. Participants who had completed TPT or were taking TPT at the time of the interview demonstrated more knowledge and understanding about TB preventive treatment. Clinicians recognised that familiarity with TPT might vary and developed programmes and support systems to increase understanding among potential recipients. In Zimbabwe, participants were offered additional support and education through regular support group sessions where participants and clinicians gathered and shared experiences and information.

They decided that it was best to have an adolescent day that young adolescents would gather to share a little bit of ideas and stuff then they give us our medication. We discuss a lot, we talk about SRH [sexual and reproductive health], terms of disclosure, how you disclose to your partner, the importance of adhering to our ARV and TPT medication, eating nutritious foods, we discuss a lot. (20–30 years old female, completed 3HP, Zimbabwe)

In Ethiopia and South Africa, some participants educated themselves further on TPT after being introduced at the facility level. South African participants noted that information provided by the clinicians was minimal, thus they relied on the internet to get further information on TPT and why they were prescribed.

Well with regards to IPT, I was never introduced that much. The nurse that was assisting me that day did not talk much about the IPT but because I like to read a lot, I googled. So, I found out that because I had COVID-19 so I was almost exposed to TB, so I think that is the reason that she gave me pills to prevent TB. (30–40 years old female, still on IPT, South Africa).

Through the knowledge acquired about TPT at the facilities and lived experiences of close kin, participants were aware of the benefits of TPT. Some participants shared benefits that they believed were TPT-related based on changes that they had experienced or heard about from others.

What I learnt is that to prevent is always good… As you can see that even flu did not manage to infect me, also having the continuous headache that I used to have been now a thing of the past. I will be acknowledging that the TPT is good for us so I learnt that I should always make sure I have prevented. (20–30 years old male, completed 3HP, Zimbabwe) (table 3)

**Table 3:** IMB themes and illustrative quotes

IMB domains	Key themes	Illustrative quotations
Information	Knowledge of TPT and understanding of TPT benefits	*What I learnt is that to prevent is always good… so I learnt that I should always make sure I have prevented*. (20–30 years old male, completed 3HP, Zimbabwe)
	Confusion about the role of TPT in the absence of TB disease	*I did not take it (TPT))because I was examined and got a negative (TB)) test result. They told me there is no disease*. (70–80 years old female, refused TPT, Ethiopia)
Motivation	Prior TB/TB treatment experiences	*First, I was infected with TB disease unknowingly and I did not apply the preventive measure. I did not know about TPT, was that the reason I was like that and severely sick? Finally, after I get to know it (TPT*)*, I decided to take the drug because I believed the drug would help me prevent*. (Female, age not recorded, completed IPT, Ethiopia)
	Engagements with HCWs	*They do everything they can in the health facility. They will help you and advise you. If there is a problem, they give us advice and a solution*. (50–60 years old male, still on IPT, Zimbabwe)
	Social support and remaining healthy for children	*I took it (3HP) because I trusted that I won’t get sick, because I am the breadwinner at home, and then I wanted to also prevent TB because I did not want to stay at home, reason being my children will suffer, and have no one who will bring anything to the table*. (40–50 years old female, discontinued 3HP, South Africa)
Behavioural approaches	Adoption of adherence strategies	*Since I am taking ART, I did not want to have to be taking a lot of medication at the same time so in the morning I would take the one for every day (pyridoxine) and then in the evening if it is a Tuesday, I take my TPT that one for once a week (3HP) and then my ART at the same time*. (20–30 years old female, completed 3HP, Zimbabwe)
	Fear of pill burden and contraindications	*The one thing I feared most was that they were going to clash with the current medication that I am taking. I felt as if they will make me very ill to the point where I will be hospitalised. (20–30 years old female, discontinued 3HP, South Africa*)
	Side effects	*It’s because the side effects were too harsh, I cannot say it was not bad but the fact that it was too much, so it demoralised me*. (20–30 years old female, discontinued 3HP, Zimbabwe)

ART, Antiretroviral therapy; HCW, Healthcare worker; 3HP, high-dose rifapentine 900 mg plus isoniazid 900 mg weekly for 3 months; IMB, information-motivation-behavioural skills; IPT, Isoniazid preventive therapy; TPT, tuberculosis preventive treatment.

One participant used her lived experience as the foundation of her understanding of how TPT worked and its effectiveness:

I have learnt that TPT does work. When my husband goes to work at the mine, he is not getting infected with TB. We just hear that his friend got infected with TB and that the other one has TB as well and one of them died. My husband goes to the same mine, but he has never been infected with TB and so that is when I realized that TPT does work. (20–30 years old female, completed IPT, Zimbabwe)

Participants attributed additional benefits to TPT, even though not medically proven. These included prevention from the general cold or influenza, continuous headaches, boosting of the immune system, and protection from other diseases.

I found it [3HP] useful. It protects you from co-morbidities and is important for TB. It does not affect the liver; it means that it protects it; it is useful for related diseases. It is good; it will not affect the lungs. You should just not forget and take the medicine… (30–40 years old female, completed 3HP, Ethiopia)

#### Confusion about the role of TPT in absence of TB disease or in addition to ART

Some participants, particularly those who refused to initiate TPT, expressed views about TPT that contradicted the goals of the treatment. For example, some participants shared that there was no reason to take TPT if one did not have TB disease or did not show any signs of TB. For one participant, this belief was endorsed by his healthcare provider:

I did not take it because I was examined and got a negative [TB] test result. They told me there is no disease. Hence, I just take ART drugs, one pill every night. They [HCWs] told me, I should not take it. I do not have TB disease. (70–80 years old female, refused TPT, Ethiopia) (table 3)

A participant in Ethiopia expressed that his health was in good condition due to the ART he was taking, thus he did not see the need for TPT.

The reason why I didn’t take it is, first of all, that I have a little bit more understanding now. My immune system is very developed because I take ARVs. I don’t believe that I will get TB. At the moment, I protect myself to a certain extent. First, the virus has been suppressed and second, why would I add side effects by increasing the amount of medicine? I don’t want to take the medicine because I have nothing. (40–50 years old male, Refused TPT, Ethiopia)

Similarly, a participant in South Africa believed that TPT and TB treatment worked in the same way and that there was no need for TPT in addition to ART. The participant also believed that TB treatment would protect him even after stopping the medication.

I think it is the same thing [TB treatment and TPT], the ones that prevent TB the way I see things it means they are pills that make TB not to come back all together. I did not want to take them [TPT] because I have taken the other ones for TB before. The way I see things like I have said before that when you take the ones for HIV, they will make you to be in a good stage so no need… (40–50 years old male, Refused TPT, South Africa)

A participant in South Africa shared how he relied on cannabis to protect himself from contracting TB, thus not needing TPT.

Yeah, I received them [ART and TPT] at the same time. To be honest, I am someone since I was born, I have never taken pills like Grandpa, Disprin [medication for pain relief] you see. So, I am someone who uses I call them international help you see, so it helps for TB prevention… It is ganja, it’s marijuana. But I must take ARVs for HIV so that they can work and for me to be stronger than before you see. You drink it, boil it, or smoke it or whatever, it is good for prevention…yeah, I am sure 100 percent sure, the one who cooks, boils, and takes it, good it is good it beats TB like nothing. (40–50 years old male, refused TPT, South Africa)

### Theme 2: motivation

#### Prior TB/TB treatment experiences

For participants who had been previously diagnosed with TB and initiated on TB treatment, the prior illness experience created a fear of reinfection with TB, which motivated uptake of TPT. Participants described wanting to prevent recurrence and maintain their health:

I wanted to prevent myself, that’s what motivated me. So that I can be prevented so that I cannot go back to having TB again…not be infected again with TB, so that I don’t get sick again. (30–40 years old female, still on IPT, South Africa)

Participants also valued TPT as a protective measure, recognising its role in preventing future TB infection:

First, I was infected with TB disease unknowingly and I did not apply the preventive measure. I did not know about TPT, was that the reason I was like that and severely sick? Finally, after I get to know it [TPT], I decided to take the drug because I believed the drug would help me prevent. (Female, age not recorded, completed IPT, Ethiopia) (table 3)

Some participants expressed how TPT was more manageable than TB treatment, particularly in terms of dosage and frequency, making adherence easier.

For TB treatment I used to take 14 tablets how then can only 4 tablets [of 3HP] become difficult for me to take it’s not possible. The TB ones we would take 14 tablets everyday but for TPT it’s only every Tuesday not every day. Only 4 every Tuesday cannot be difficult. If we do not get them, we might end up dying. (50–60 years old male, still on 3HP Zimbabwe)

Participants noted that pill burden and dosing frequency influenced their experience with TPT, with differences between 10-pill 3HP, 3-pill fixed-dose combination, and daily isoniazid affecting perceived ease of adherence and motivation.

#### Social support and remaining healthy for children

Social support from family and healthcare workers was identified as a key motivator for initiating TPT.

What I can say is that she [the sister] encouraged me take them since I didn’t want to. Yes, I feared to react and adding another condition on top of the HIV I already have. But all she did was to encourage me to take the medication, she told me that these ones are good so I should stay prevented from TB. (40–50 years old female, completed 3HP, Zimbabwe)

Some participants expressed the need to maintain good health for the sake of their children and families, who relied on them financially.

I took it [3HP] because I trusted that I won’t get sick, because I am the breadwinner at home, and then I wanted to also prevent TB because I did not want to stay at home, reason being my children will suffer, and have no one who will bring anything to the table. That is why I took the treatment, I took it because I was protecting myself… (40–50 years old female, discontinued 3HP, South Africa) (table 3)

Social support and the family situation were identified as key to ensuring adherence to medication. Participants shared experiences of how family and friends encouraged them to continue taking their medication and assisted where necessary.

My wife is also on the same page (taking ART and TPT), at 8 o’clock, you know how women are, she takes hers and she also gives me mine. Unless, I go out to my places, but when I come back, she gives them to me… that is the first priority she helps with. (40–50 years old male, completed 3HP, South Africa)

#### Engagements with HCWs

In Ethiopia and Zimbabwe, participants mostly shared that the positive engagements they had with HCWs and the clinical services they had received encouraged them to initiate and persist on TPT.

They do everything they can in the health facility. They will help you and advise you. If there is a problem, they give us advice and a solution. They say that if you come here and consult us, we will find a solution. There is not as much problem with the health centre. They are good. They treat you well. They gave us counselling services regarding using medicine to fight the disease and taking care of ourselves. (50–60 years old male, still on IPT, Zimbabwe) (table 3)

Ethiopian participants further described sharing concerns about side effects and their TPT journeys with clinicians without fear.

I came here and told them about it [side effects], they told me that it is because I am new to take this drug, it is how everyone feels at the beginning and they were saying, ‘don’t worry.’ They just give me advice. (30–40 years old female, completed IPT, Ethiopia)

For some South African participants, the negative engagements with HCWs and bad service received from health facilities was largely linked to the discontinuation of TPT.

The nurses are rude…even when you tell them the pills are not treating me well they will just yell at you, I don’t know why they yell, they don’t have to yell at you they must just explain to you that, ok sometimes the side effects of these pills they are like this… that is why I did not tell them I just kept quiet and said to myself maybe it shall get better. Then I stopped. (20–30 years old female, discontinued 3HP, South Africa)

#### Religious beliefs

For some participants in Ethiopia, religion and faith were an instrumental motivator for either initiating or not initiating TPT. A participant attributed her lack of side effects to God.

Yes, as a human I had fear of taking the drug [IPT]. What would I do, if something happened to me after taking this drug? I do not have anyone else besides me. I had no one to help and support me. I finally decided, God, I am doing this with your support and mercy. Even though the health care providers told me about the side effects of taking the drug I did not experience them because of God. I did not speak with people, but with the almighty God. (Age not recorded, female, completed IPT, Ethiopia)

A participant who refused TPT shared that she was not afraid of contracting TB because God would protect her as had been the case all along.

I do not have fear; all those years I have full confidence in the protection of God. I also protect myself as far as I can. I was taken care of myself; I did not have fear of TB disease. I had no history of TB infection and I do not have a history of taking TB treatment medication. (40–50 years old female, refused TPT, Ethiopia)

#### Food insecurity

In Ethiopia and Zimbabwe, we found that some participants were also concerned about the additional burden of food insecurity and how that impacted the treatment process.

If I am infected with TB, I will take it [TPT] I do not take it arbitrarily; I take it after being tested…It is hard, taking the additional drug with ART drugs is difficult, it is difficult with such challenging life-supporting issues, it needs adequate food, you can’t simply swallow the drug eating any food like Shiro wat [local name for chickpea stew]. (70–80 years old female, refused TPT, Ethiopia)

A participant who had been instructed to take the medication with food shared that he did not always have access to food.

The issue of taking the pills with food, it is a challenge. Let us say that you struggle to get food, some days you can go without food what then do you do with these pills… I was asking myself that they are saying you should take the pills with food, you are not supposed to skip any dose, what if I fail to find food at the time am supposed to take the pill, what do I do. (50–60 years old male, discontinued 3HP, Zimbabwe)

### Theme 3: behavioural approaches

#### Adoption of adherence strategies

To ensure completion and persistence of TPT, participants shared various adherence strategies and behavioural approaches they adopted. These included setting alarms, linking medication time with TV shows and using medication pill boxes offered by the study team. For others, having been on ART for a long time made it easy for them to remember to take their medications. Participants also adopted strategies to manage side effects and pill burden.

Since I am taking ART, I did not want to have to be taking a lot of medication at the same time so in the morning I would take the one for every day (pyridoxine) and then in the evening if it is a Tuesday, I take my TPT that one for once a week (3HP) and then my ART at the same time. (20–30 years old female, completed 3HP, Zimbabwe) (table 3)

#### Fear of pill burden and contraindications

Fear of pill burden and contraindications of medications was common among participants in South Africa. This was noted as a major concern leading to non-initiation of TPT.

It’s just a matter of being scared of having more than one, or rather more than two ailments in my body like I am already positive with both epilim [an epilepsy medication] and ARVs treatment now having to take another treatment…I don’t need extra [pills] my body is carrying too much now you know ARVs, epilim too much for one person so now if I have to add this one [TPT]. Ah! (50–60 years old female, refused TPT, South Africa)

The number of pills was also a common reason contributing to the discontinuation of TPT.

I took it for two weeks only…unless they change the whole pill, I don’t know but I don’t see myself taking it unless they change because number one those pills are plenty, my God! 10 pills! So, if they can reduce the amount of tablets maybe I would [take 3HP again]… combine them into one thing or I don’t know…make them 5 or less than, may be yeah and less stronger. (20–30 years old female, discontinued 3HP, South Africa)

In addition to the side effects, some South African participants also shared concerns with the TPT drugs interfering with other chronic medications they were already taking. In addition to living with HIV, some participants shared experiences of living with other comorbidities.

The one thing I feared most was that they were going to clash with the current medication that I am taking. I felt as if they will make me very ill to the point where I will be hospitalized. The TPT is very strong I won’t lie, it’s very its super strong. (20–30 years old female, discontinued 3HP, South Africa) (table 3)

#### Side effects

For participants who had discontinued TPT, overall the primary reasons for discontinuation were linked to experienced side effects. These participants shared that anticipating or experiencing side effects discouraged them from continuing with the treatment regimen.

It’s because the side effects were too harsh, I cannot say it was not bad but the fact that it was too much, so it demoralised me. I do not know if maybe there was a way that if someone takes those tablets and develops side effects there should be people on standby who will explain to them that it is normal, and you will be fine, or the patient prescribed some medication to deal with the side effects so that someone does not get depressed. (20–30 years old female, discontinued 3HP, Zimbabwe) (table 3)

Even though some participants had not taken TPT, some shared concerns with developing side effects due to the treatment. Some of these were witnessed while others were perceived side effects of TPT.

Whenever I hear about TPT what comes to my mind is that I will fall sick. I will get bedridden due to illness after taking TPT. That is what comes to my mind. I have also heard that TPT causes side effects and causes a lot of hunger. Getting very dark in complexion, having swollen legs, and peeling off skin. (40–50 years old male, refused TPT, South Africa)

Some shared how they had experienced side effects with their ART, thus feared that the TPT would also have side effects.

The after effects because we all know that every drug has its after effects that is the only part that I am sceptical about. What would happen if it doesn’t go well with my system because with others you find loss of appetite and other things like smelly urine, smelly discharges that’s what I have experienced with ARVs, so I am worried what will be the after effects of this too [TPT], that is my only concern. (50–60 years old female, refused TPT, South Africa)

## Discussion

Consistent with the Informational-Motivation-Behavioural Skills (IMB) model, participants’ accounts highlighted the central role of information in shaping experiences of TPT. Understanding of TPT, including its purpose, benefits and potential side effects emerged as an important factor associated with decisions to initiate, continue, refuse or discontinue treatment. Our findings have also demonstrated that TPT education on side effects management, drug-drug interactions and pill management is appreciated when provided at clinic or community level. Investing in provider training, patient-centred job aids and community engagement strategies may improve TPT knowledge and uptake by strengthening patient education, addressing misconceptions and supporting treatment adherence. Previous studies have shown that TB literacy and counselling interventions can improve patient engagement, treatment uptake and self-efficacy, while community-based approaches have been associated with improved TPT adherence and treatment completion among PLHIV.^30^,^32^ Experiences of participants in Zimbabwe show that peer support and education have a positive influence on health behaviours. In addition to peer support and education, we recommend interactive counselling where patients can have a platform to address concerns and seek information to aid their treatment journeys. Through the experiences of participants in Ethiopia, whom religion and faith was central to their lived experience, integrating religious leaders and faith-based communities to the clinical services have yielded positive outcomes.^33 34^ Re-enforcement of motivation from clinic-level, enhancing adherence strategies and attending routine care may increase uptake and completion of TPT. Lastly, consistent messaging from healthcare workers should be aligned with local social and cultural contexts. We recommend further patient-centred approaches in implementation of interventions through understanding end user experiences and preferences.

Among those who completed TPT, the common reasons were related to the perceptions and information participants had about TB and TPT. Participants demonstrated an understanding of TB disease, the relationship between TB and HIV and the protective power of TPT. This understanding benefited behaviours in line with completion of TPT, consistent with prior literature.^35^,^37^ Literature demonstrates that patient education and engagement increases the patient’s ability to understand, seek and act on health behaviour. This can have a positive impact in the patient’s present and long-term health.^35^,^38^ Our study also identified that participants with information about TB and TPT were motivated to complete TPT, thus developed adherence strategies to ensure persistence and completion. A qualitative systematic review of factors associated with adherence to TPT among PLHIV also highlighted that patient’s understanding of and beliefs related to treatment positively influenced adherence to TPT.^39^ Our participants’ adherence strategies included setting alarms and having trackers, taking ART and TPT at the same time or reminders from the family.

In this study, experiencing side effects, pill burden and concerns with additional medications for chronic conditions were identified as key reasons for TPT discontinuation. A retrospective data review conducted in Ethiopia in 2022 showed that 23% of PLHIV who initiated TPT discontinued, and fear of side effects were identified as one of the challenges in the region.^40^ Side effects and pill burden are identified as major barriers to TPT completion across different settings.^12 40 41^ Our findings also highlight that differences in pill burden and TPT regimens influenced participant experiences and adherence across countries. For example, participants receiving 10-pill 3HP often reported greater concern about pill load compared with those on 3-pill fixed-dose combination or daily INH, although all regimens were generally considered easier than full TB treatment. In our study, participants also expressed a need for prior knowledge or education about potential side effects of taking TPT, a finding consistent with other studies conducted in South Africa.^10 42^ In line with the IMB model, the lack of adequate information about the potential side effects discouraged participants from continuing with TPT or seeking medical assistance. Therefore, for this category of participants, there was no clear motive for persisting on medication, thus they discontinued. Additionally, concerns with taking additional chronic medication were linked with lack of knowledge on drug-drug interactions. This viewpoint is consistent with a study conducted in South Africa, among PLHIV, highlighting that despite recognition of TPT’s importance, concerns regarding side effects and treatment burden underscore the need for enhanced patient education, strengthened healthcare communication, and proactive framing of TPT to improve uptake.^43^ For our participants who were already established on ART, and in some cases taking other chronic medications, the addition of TPT to their treatment journeys posed fears about the negative drug-drug interactions.

Participants who refused to initiate or discontinued TPT attributed this choice to lack of understanding of the role of TPT in the absence of TB disease or symptoms, fear of side effects, pill burden and fear of contraindications. Concerns regarding pill burden were predominantly reported by South African participants receiving 3HP. Unlike in Ethiopia and Zimbabwe, where 3HP was prescribed as a 3-pill fixed-dose combination, the regimen in South Africa comprised 10 pills, including pyridoxine. For participants who did not initiate TPT, lack of information or misinformation about TPT was a common reason for non-initiation. This finding is consistent with existing literature, showing inadequate understanding of TPT as one of the key barriers to TPT acceptance and completion among PLHIV.^11 12 41^ A study describing IPT perceptions and experiences in communities in South Africa found that understanding the realities of end users was crucial for the success of TB control efforts.^42^ Our study highlighted that PLHIV commonly live with other comorbidities and dealt with numerous medications daily. Due to this, continuous education about medications and how they interact could enhance understanding and result in positive beliefs and motivations for TPT uptake.^35 38^ Substance use was reported as a potential barrier to TPT initiation in this study. Previous studies suggest that individuals who use substances may be more likely to experience reduced health-seeking behaviour, lower adherence to medication or increased concerns about side effects or interactions, particularly when managing multiple comorbidities.^44 45^ Additionally, food insecurity emerged as a concern and reason for refusal or discontinuation of TPT among some participants in Ethiopia and Zimbabwe, who described the added burden it placed on the treatment process. This aligns with evidence from a scoping review on HIV, TB and food insecurity in Africa, which highlights the negative effects of food insecurity on health outcomes, including treatment non-adherence.^46^

This study has several important strengths. First, it draws on a large qualitative dataset of 141 in-depth interviews conducted across three countries in sub-Saharan Africa, providing diverse perspectives from different health system and sociocultural contexts. The inclusion of participants across the full TPT cascade—those who completed treatment, were currently receiving TPT, discontinued treatment or declined initiation—enabled a comprehensive understanding of factors influencing TPT uptake and persistence. The use of the IMB model provided a strong conceptual framework to guide data collection and analysis, strengthening the interpretation and practical relevance of the findings. Methodological rigour was enhanced through purposive sampling, multilingual interviews, verbatim transcription, iterative coding by multiple researchers and adherence to established qualitative reporting standards. Finally, the study was embedded within a real-world implementation programme and included stakeholder engagement, enhancing the relevance of the findings for policy and programme implementation in similar settings. However, this study has some limitations. First, the study population consisted of participants purposively recruited from the main Opt4TPT study who had been offered TPT, which may limit the generalisability of our findings to populations not involved in the main study. Second, participation in the main study and any associated incentives may have introduced selection bias at the study enrolment stage, and may also have influenced participants’ engagement with TPT, potentially increasing adherence or persistence. Third, participant responses may be subject to recall and information bias, particularly when reflecting on prior experiences with TB or TPT. Fourth, project or facility-level characteristics, including variations in staffing, counselling practices, and TPT delivery, may have shaped participant experiences and perceptions. Finally, we did not analyse code frequencies or generate quantifiable indicators from our thematic analysis; our findings are presented qualitatively based on recurring themes and illustrative quotes. While this approach captures the richness of participants’ perspectives, it does not provide numeric estimates of theme prevalence. Future research could complement these qualitative insights with quantification of code frequencies to enhance empirical grounding and analytical rigour.

## Conclusions

Participants identified a variety of reasons within each dimension of the IMB model which influenced behaviour, that is, TPT initiation and persistence or TPT non-initiation or discontinuation. These reasons are modifiable through clinic and community-level interventions such as targeted health messaging, peer support and education, interactive counselling sessions, and integrating community structures that participants occupy into the clinical interventions. Addressing these reasons may enhance TPT uptake and completion. In addition, addressing substance use through integrated care models and providing tailored counselling may support greater TPT uptake in this high-risk population. We recommend adopting patient-centred approaches in implementation of interventions, grounded in a deep understanding of end-user experiences and preferences.

## Data Availability

Data are available upon reasonable request.
